# P03-08 Do sports and PA offer a special arena with particularly good opportunities to work with the personal development of young people? - A study of young people's personal developing opportunities on sports-based independent boarding schools in Denmark

**DOI:** 10.1093/eurpub/ckac095.044

**Published:** 2022-08-29

**Authors:** Sofie Morley, Lise Maria Elkrog-Hansen, Lars Breum Christiansen

**Affiliations:** Department of Sports Science and Clinical Biomechanics, University of Southern Denmark, Odense, Denmark

**Keywords:** personal development, sports, physical activity, school

## Abstract

**Background:**

Independent boarding schools are a unique Danish type of residential setting for young people between the ages of 14 to 18. Distinguishing independent boarding schools from Danish public schools is, the independent boarding schools' distinct obligation to promote the ‘personal development' of the students. Approximately half of the 242 Danish independent boarding schools have an explicit focus on sport and physical activity (PA). Thus, it is interesting to investigate if sports-based independent boarding schools offer an arena with particularly good opportunities to work with the personal development of young people?

**Methods:**

In March 2019 a survey was distributed to 1020 students at three participating sports-based independent boarding schools. This survey has since been developed further and in March 2022, it will be distributed nationwide to approximately 120 schools, with the scope of reaching approximately 18.000 students. To gain insights in regard to the aim of the study, the survey will collect quantitative data on sociodemographics of the students, motives for choosing independent boarding school life, the students' perception of the pedagogical practice in sports and PA lessons, the students' perceived personal development, and the students' well-being.

**Results and conclusion:**

The study will take place in March 2022 and thus data and conclusions will be presented at the conference.

